# Alemtuzumab in Multiple Sclerosis: Short- and Long-Term Effects of Immunodepletion on the Peripheral Treg Compartment

**DOI:** 10.3389/fimmu.2019.01204

**Published:** 2019-06-04

**Authors:** Jürgen Haas, Cornelia Würthwein, Mirjam Korporal-Kuhnke, Andrea Viehoever, Sven Jarius, Tobias Ruck, Steffen Pfeuffer, Sven G. Meuth, Brigitte Wildemann

**Affiliations:** ^1^Division of Molecular Neuroimmunology, Department of Neurology, University Hospital Heidelberg, Heidelberg, Germany; ^2^Department of Neurology and With Institute for Translational Neurology, University of Muenster, Muenster, Germany

**Keywords:** regulatory t cells, human, immunodepletion, alemtuzumab, multiple sclerosis

## Abstract

Treatment with alemtuzumab is followed by an early increase in Treg frequencies. Whether naïve and memory subsets are differentially affected and how depletion influences dysfunctional MS-Treg is unclear. In this study, we analyzed the effect of alemtuzumab on regulatory T-cells (Treg) in patients with multiple sclerosis (MS). For this purpose 182 blood samples from 25 MS patients were taken shortly before treatment and serially for up to 24 months after two alemtuzumab cycles. We studied Treg by flow cytometry (quantitation, phenotypical characterization), real-time polymerase chain reaction (T-cell receptor (TCR) excision circles [TREC] content), CDR3-spectratyping (clonal distribution), and proliferation assays (suppressive function). CD52-mediated cytolysis of Treg and conventional T-cells was determined by a complement-dependent cytolysis assay. Our studies revealed that 1 week post-alemtuzumab, Treg were depicted at constant frequencies among CD4^+^ T-cells. In contrast, Treg frequencies were massively increased at month 1. Post-depletional Treg exhibited a CD45RO^+^ memory phenotype, a skewed TCR repertoire, and contained minimum TREC numbers. Naïve Treg, thymic markers, and TCR-variability commenced to rise after 6 months but did not attain baseline levels. *In vitro*, Treg exhibited higher susceptibility to lysis than Tcon. Treg suppressive function constantly increased within 1 year when co-cultured with syngeneic T-cells, but remained stable against allogeneic T-cells from normal donors. Our findings suggest that (1) Treg are not spared from alemtuzumab-mediated depletion and thymopoiesis does not considerably contribute to long-term recovery, (2) either homeostatic proliferation and/or conversion from residual Tcon contributes to Treg expansion during the early post-treatment phase (3) the enhanced inhibitory effect of Treg following alemtuzumab is due to altered composition and reactivity of post-depletional Tcon.

## Introduction

Alemtuzumab, a humanized anti-CD52 monoclonal antibody (mAb), has consistently been shown to provide higher efficacy than the baseline disease-modifying agent interferon-beta1 and is approved in more than 65 countries for use in patients with active relapsing–remitting multiple sclerosis (RRMS) (1–6). Treatment with alemtuzumab leads to a depletion of circulating B- and T-lymphocytes, which is followed by reconstitution and rebalancing of the immune system, resulting in prolonged RRMS disease suppression. This process is characterized by differing alterations in numbers and proportions of different lymphocyte subsets (7–9). In contrast to the clear-out of conventional CD4^+^ T-cells (Tcon), an increase in frequencies of regulatory T-cells (Treg) early after initiating alemtuzumab therapy has been reported (10–14). This selective enrichment in Treg of the CD4^+^CD25^hi^CD127^low^FOXP3^+^ phenotype is of particular interest, as these cells are important regulators of the immune system (15). *In vitro*, Treg suppress Tcon immune responses in a dose-dependent manner (16), suggesting a potential impact of treatment-induced changes in the Treg/Tcon ratio. Alemtuzumab predominantly affects CD4^+^ T-cells exhibiting a CD45RO memory phenotype (10) and, thus, may also exert different differential effects on naïve and memory Treg subsets. Elucidating this aspect is important because Treg are functionally deficient in patients with MS, as we and others have previously shown (17–22). Of note, the loss of Treg suppressive properties is precipitated by contraction of CD45RA^+^ naïve and CD45RA^+^CD31^+^ recent thymic emigrant (RTE) subtypes and reciprocal expansion of memory phenotypes (CD45RO^+^) within peripheral Treg, a feature which possibly arises from premature immunosenescence (23–25). To decipher in detail the effects of alemtuzumab on the Treg compartment, we undertook a longitudinal study of 25 MS patients undergoing two cycles of treatment with alemtuzumab and assessed different parameters of Treg neogenesis and Treg suppressive function.

## Methods

### Human Samples

Our study included 25 patients with RRMS established according to the 2011 McDonald criteria (26), all of whom were scheduled for treatment with alemtuzumab, and 21 healthy donors. Alemtuzumab was administered as primary immunotherapy in 15 and as escalating treatment in 10 individuals, respectively. The mean disease duration was 6.3 years (range 2–13), the median age 32.3 years (17–53), and the median Expanded Disability Status Scale (EDDS) score 3.5 (1.0–6.5). During the observation period a total of nine relapses (year 1 post-treatment: *n* = 1, year 2 post-treatment: *n* = 8) occurred in eight patients, and one patient developed secondary autoimmune thyroiditis within 10 months after the second alemtuzumab administration. All patients were recruited in the Department of Neurology, University Hospital Heidelberg. Samples from 14/25 patients were repeatedly assessed over a period of 12 months after the second cycle of treatment. A total of 182 peripheral blood specimens (50–70 ml of EDTA blood and 10 ml of serum) were taken, directly before infusion and repeatedly thereafter (at day 7 and months 1, 3, 6, and 12 after each cycle). Plasma and serum samples were immediately stored at −70°C. The protocol was approved by the University Hospital Heidelberg ethics committee; all individuals gave written informed consent.

### Cell Separation

Peripheral blood mononuclear cells (PBMCs) were isolated by Ficoll-gradient centrifugation (Biochrom, Berlin, Germany). Total Treg and Tcon were highly enriched from freshly isolated PBMCs by means of immunomagnetic separation utilizing Dynabead technology as previously described (19, 27, 28).

### Flow Cytometry

**Figure 1:**
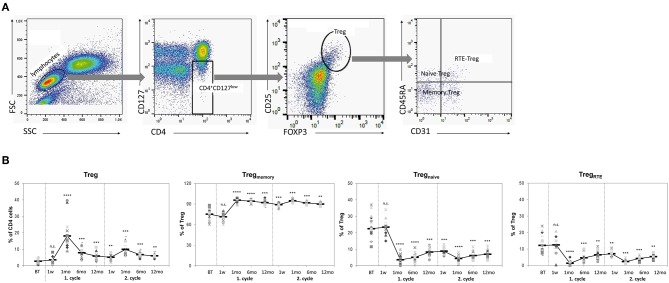
Treg repopulation is dominated by memory phenotypes. **(A)** Flow cytometry: Gating strategy for detection of Treg and Treg subtypes. Stained PBMCs were first gated on CD4 and CD127. CD4^+^CD127^low^ T cells were analyzed for co-expression of FOXP3 and CD25 identifying CD4^+^CD127^low^CD25^high^FOXP3^+^ Tregs. Tregs were then analyzed for expression of CD45RA and CD31 to identify CD45RA^+^ naive Tregs, CD45RA^−^ memory Tregs and CD45RA^+^CD31^+^ recent thymic emigrant (RTE)-Tregs. **(B)** Frequencies of Treg and Treg subsets in peripheral blood samples obtained from 25 RRMS patients before treatment (BT) and up to 24 months after two cycles of alemtuzumab. Each symbol represents an individual patient. Means are indicated by bold line. Statistical significances in differences of each time point compared to BT as determined by two-tailed non-parametric one-sample Wilcoxon sign rank test are indicated (n.s. = non-significant; ^**^p < 0.01; ^***^p < 0.001; ^****^p < 0.0001).

For quantitative and phenotypic characterization of Treg, Treg subsets, and Tcon subsets we used established multicolor flow cytometry protocols and gating strategies (19, 24, 29, 30). In short, freshly isolated PBMCs were immediately stained with a panel of mAbs specific for human Treg markers (anti-human CD4, CD45RO, CD45RA, CD31, CD127 [BD Pharmingen]; anti-human CD25 [Miltenyi Biotech]; anti-human FOXP3 [eBioscience]) (Figure 1A), or alternatively for CD4, CD25, CD45RO, and CCR7 (**Figure 4A**), and then analyzed with a FACS Calibur™ cytometer using CellQuest™ software (BD Biosciences). To determine surface expression of CD52 on Treg and Tcon, PBMCs obtained from five healthy donors were co-stained with mAbs specific for Treg and Tcon and naive or memory phenotypes (see above) and a mAb specific for human CD52 (Alexa Fluor®488 conjugated, BD Biosciences). Mean fluorescence intensities (MFI) for CD52 were then determined in gated Tcon and Treg and in Tcon and Treg subtypes. Detection and quantification of Treg with two different T-cell receptor (TCR) Vα chains were achieved using a previously described protocol (29, 31). Briefly, fresh PBMCs were stained for Treg markers (see above) and with mAbs specific for human TCR-Vα2 (FITC-conjugated, Pierce) and Vα12 (APC-labeled with Zenon Mouse IgG Labeling Kit, Molecular Probes), identifying Vα2^+^, Vα12^+^ as well as Vα2^+^Vα12^+^ (double-positive) cells in gated Treg to calculate proportions of dual TCR cells as described (29, 31). To quantify alemtuzumab-induced cytolysis *in vitro*, Tcon or Treg tested in complement-dependent cytolysis assays were washed and then co-stained with mAbs specific for Treg and Tcon subtypes (see above) and propidium iodide (PI). Cytolysis rates for each subtype were calculated from the proportions of PI-positive cells after exposure to alemtuzumab minus the proportions of PI-positive cells after exposure to a control antibody.

### Cell Pro-Liferation Assay

To measure inhibitory capacities, patient- or donor-derived Treg were tested using *in vitro* proliferation assays against (a) syngeneic Tcon (obtained from the same patient) and (b) allogeneic Tcon (obtained from a frozen pool of Tcon from eight healthy donors). An amount of 4 × 10^4^ Tcon (either syngeneic or allogeneic) was incubated alone or in coculture with 1 × 10^4^ Treg (Treg/Teff ratio 1:4) and polyclonally activated by adding soluble anti-CD3 (1 μg/ml) and anti-CD28 mAbs (1 μg/ml). After 4 days, the cells were pulsed for 16 h with 1 μCi of ^3^[H]-thymidine per well. After harvesting, T-cell proliferation was measured with a scintillation counter.

### Complement-Dependent Cytolysis Assay

To screen for possible differences in alemtuzumab effects on Tcon and on Treg subsets, 2 × 10^5^ freshly isolated total Treg or Tcon obtained from five healthy subjects were incubated for 3 h at 37° with 10 μg/ml alemtuzumab (Genzyme) or control human IgG (Jackson ImmunoResearch) and 10% normal human complement (Quidel) in X-vivo 15 medium (BioWhittaker) supplemented with 10% FBS (Biochrom). After the incubation period, cells were washed and re-suspended in medium for subsequent flow cytometry analysis.

### Quantification of T-Cell Receptor Excision Circles

Total DNA was extracted from freshly isolated Treg (1–3 × 10^5^ cells) using a QIAmp Blood Mini Kit (Qiagen) according to the manufacturer's protocol. Numbers of T-cell receptor-excision circles (TRECs) were determined by real time PCR as described elsewhere (32) and expressed as TRECs/10^6^ Treg.

### CDR3 Spectratyping

First, total RNA was isolated from freshly isolated Treg (2–5 × 10^5^ cells) using a Rneasy Mini Kit (Qiagen) according to the manufacturer's protocol and then converted to cDNA using a SuperScript® III First-Strand Synthesis System (Invitrogen). For real-time PCR detection of all 24 TCR Vβ subfamilies (33, 34), 100 ng of cDNA was set in eight parallel PCR reactions, each containing three different Vβ specific primer sets and a 6-FAM-labeled C-primer for subsequent CDR3 spectratyping. Size distribution of 6-FAM-labeled PCR products was determined by laser-induced capillary electrophoresis with an automated DNA analyzer A310 and GeneScan software (Applied Biosystems). The complexity score within a Vβ subfamily was determined by counting the number of peaks per subfamily. Normal transcript size distribution consists of eight peaks for each V subfamily (35). A complexity score (CS) was calculated as the sum of scores of all 24 TCR Vβ subfamilies.

### Statistical Analysis

To determine whether differences in cell counts, inhibitory capacities, TREC levels, and TCR diversities were statistically significant, we performed non-parametric one-sample Wilcoxon sign rank tests using a two-tailed distribution with paired samples. No analysis of variance [ANOVA] was carried out, because data sets were not complete for all patients and all time points analyzed. A *p* value of ≤ 0.05 was considered to show be statistically significant difference in a descriptive sense. Two-sided *t*-tests and paired *t*-tests were used to compare normally distributed samples (cytolysis assay, CD52 expression). Again, a *p* value of ≤ 0.05 was considered to be statistically significant.

## Results

### Treg Recovery

Shortly after immune cell depletion by alemtuzumab (week 1) a concomitant reduction in numbers of circulating CD4^+^ T cells and Treg (2 ± 2/μl [Treg cell count]), was observable resulting in constant relative proportions of Treg (3.5 ± 2.4% [of CD4^+^ T cells]) among circulating CD4^+^ T-cells compared with baseline (19 ± 6/μl, 2.7 ± 1.5%; *p* = 0.008 and 0.285). In contrast, Treg numbers had recovered to 11 ± 5/μl at 1 months, translating into a massive increase in percentages among CD4^+^ T cells (18.1 ± 9.3%; *p* = 0.022 and < 0.0001). Treg expansion then decreased, but proportions remained consistently higher at baseline throughout year 1 and year 2 (Figure 1B; Table 1; Figures S1, S2). At month 1, almost all Treg exhibited a CD45RO^+^ memory phenotype (Treg_memory_). The dominance of Treg_memory_ persisted throughout the study period, and CD45RO^+^ Treg accounted for ≥ 90% of CD4^+^ T-cells until month 12 of each treatment cycle (Figure 1B; Table 1). In contrast, frequencies of Treg with a naïve or RTE phenotype (Treg_naï*ve*_, Treg_RTE_) were barely detectable one month post-treatment and slowly recovered thereafter without returning to baseline levels. Whereas, total Treg at baseline comprised on average 23 and 12% Treg_naive_ and Treg_RTE_, Treg_naive_ accounted for 8% (*p* < 0.001) and 7% (*p* < 0.001) of T-cells and Treg_RTE_ for 6% (*p* < 0.01) and 5% (*p* < 0.01) of circulating CD4^+^ T-cells after 12 and 24 months, respectively (Figure 1B; Table 1). Hence, these observations indicate that Treg are lysed together with other CD4^+^ T-cells and—in response to treatment-induced lymphopenia—then either expand to preferentially acquire a memory phenotype and/or are converted from post-depletional Tcon. Together with the decline in frequencies of Treg_RTE_, these changes—similar to what has been reported for CD4^+^ T-cells—do not favor replacement of Treg by thymic neogenesis.

**Table 1:** Flow cytometry and PCR data obtained from 25 MS patients before and after two cycles of alemtuzumab.

		**Treatment cycle 1**				**Treatment cycle 2**			
	**Before therapy**	**1 week**	**1 month**	**6 months**	**1 year**	**1 week**	**1 month**	**6 months**	**1 year**
Treg	2.7 ± 1.5 (0.6–5.2)	3.5 ± 2.4 (0.9–8.4), *p* = 0.2849	18.1 ± 9.3 (8.0–39.6), *p* < 0.0001	7.9 ± 3.4 (3.2–14.0), *p* < 0.001	5.9 ± 2.8 (2.3–11.2), *p* < 0.001	5.1 ± 1.9 (2.0–8.0), *p* < 0.01	9.9 ± 3.5 (6.1–17.6), *p* < 0.001	6.7 ± 1.6 (4.1–10.0), *p* < 0.001	6.0 ± 1.2 (4.1–8.8), *p* < 0.01
Treg_naive_	22.5 ± 6.5 (11.5–36.9)	23.4 ± 5.1 (15.0–34.0), *p* = 0.7127	3.6 ± 3.3 (0.0–10.1), *p* < 0.0001	5.1 ± 2.2 (0.5–10.9), *p* < 0.0001	8.3 ± 2.3 (3.6–12.5), *p* < 0.001	8.8 ± 2.3 (5.6–13.0), *p* < 0.001	4.4 ± 2.2 (0.8–8.3), *p* < 0.0001	6.2 ± 2.6 (2.7–11.5), *p* < 0.001	7.0 ± 2.6 (2.9–12.2), *p* < 0.001
Treg_memory_	75.2 ± 7.1 (60.5–88.0)	71.3 ± 5.6 (60.0–79.9), *p* = 0.1174	95.5 ± 3.6 (88.1–100), *p* < 0.0001	94.0 ± 2.4 (89.0–98.1), *p* < 0.0001	92.0 ± 3.2 (85.5–99.3), *p* < 0.001	89.0 ± 3.0 (82.1–92.4), *p* < 0.001	94.7 ± 2.5 (90.3–99.8), *p* < 0.001	91.7 ± 2.3 (87.7–95.4), *p* < 0.001	89.9 ± 2.3 (86.5–94.2), *p* < 0.01
Treg_RTE_	12.2 ± 5.0 (6.0–23.9)	12.5 ± 5.3 (0.4–20.7), *p* = 0.9000	1.3 ± 1.3 (0.0–5.1), *p* < 0.0001	4.6 ± 2.7 (1.1–10.0), *p* < 0.001	6.4 ± 2.3 (2.3–11.6), *p* < 0.01	7.1 ± 2.0 (5.1–10.8), *p* < 0.01	2.4 ± 0.9 (0.9–4.7), *p* < 0.001	4.3 ± 1.2 (1.9–6.5), *p* < 0.001	5.4 ± 1.4 (3.0–7.8), *p* < 0.01
Dual-TCR-Treg	61.1 ± 4.5 (52.3–69.5)	58.9 ± 5.7 (48.0–66.0), *p* = 0.3550	45.6 ± 5.5 (37.4–53.9), *p* < 0.001	47.9 ± 3.4 (41.0–53.7), *p* < 0.001	49.9 ± 2.8 (45.1–54.0), *p* < 0.001	50.2 ± 1.5 (44.5–55.2), *p* < 0.001	46.0 ± 1.5 (41.0–52.7), *p* < 0.001	49.2 ± 2.9 (42.9–53.6), *p* < 0.001	51.0 ± 2.5 (47.0–55.6), *p* < 0.01
CDR3-complexity score	147 ± 7.1 (138–160)	n.a.	125 ± 7.6 (115–140), *p* < 0.001	128 ± 5.8 (118–137), *p* < 0.001	131 ± 5.6 (124–144), *p* < 0.01	n.a.	127 ± 5.1 (121–138), *p* < 0.01	129 ± 3.9 (124–136), *p* < 0.01	133 ± 3.8 (129–141), *p* < 0.05
TRECs	3.029 ± 846 (1,456–5,340)	n.a.	1.273 ± 448 (788–2,188), *p* < 0.0001	1.397 ± 323 (980–2,104), *p* < 0.0001	1.635 ± 359 (1,240–2,366), *p* < 0.0001	n.a.	1.388 ± 431 (907–2,205), *p* < 0.0001	1.494 ± 403 (1,054–2,243), *p* < 0.0001	1.598 ± 370 (1,193–2,267), *p* < 0.0001
CCR7^−^CD45RO^+^ TEM	14.7 ± 5.4 (9.5–18.3)	54.3 ± 15.4 (44.2–61.7), *p* < 0.001	n.a.	28.5 ± 12.3 (16.6–37.8), *p* < 0.01	n.a.	n.a.	n.a.	n.a.	n.a.
CCR7^−^CD45RO^−^ TEMRA	7.1 ± 5.1 (4.9–9.4)	17.6 ± 7.3 (12.1–22.8), *p* < 0.001	n.a.	14.3 ± 7.3 (8.5–21.6), *p* < 0.01	n.a.	n.a.	n.a.	n.a.	n.a.
CCR7^+^CD45RO^+^ TCM	29.0 ± 10.4 (18.3–27.6)	12.5 ± 6.9 (9.4.2–19.3), *p* < 0.001	n.a.	16.2 ± 6.8 (10.0–19.8), *p* < 0.01	n.a.	n.a.	n.a.	n.a.	n.a.
CCR7^+^CD45RO^−^ TCRA	38.9 ± 11.8 (31.0–46.2)	12.0 ± 4.6 (8.8.2–15.3), *p* < 0.001	n.a.	19.4 ± 4.7 (13.9–22.6), *p* < 0.01	n.a.	n.a.	n.a.	n.a.	n.a.

*Shown here are frequencies of Treg and CD4^+^ T cell subsets [mean percentages ± standard deviation, minima and maxima (in parentheses), and statistical significance (before therapy vs. each time point thereafter). n.a. = not assessed*.

### Treg Origin and Clonal Diversity

**Figure 2:**
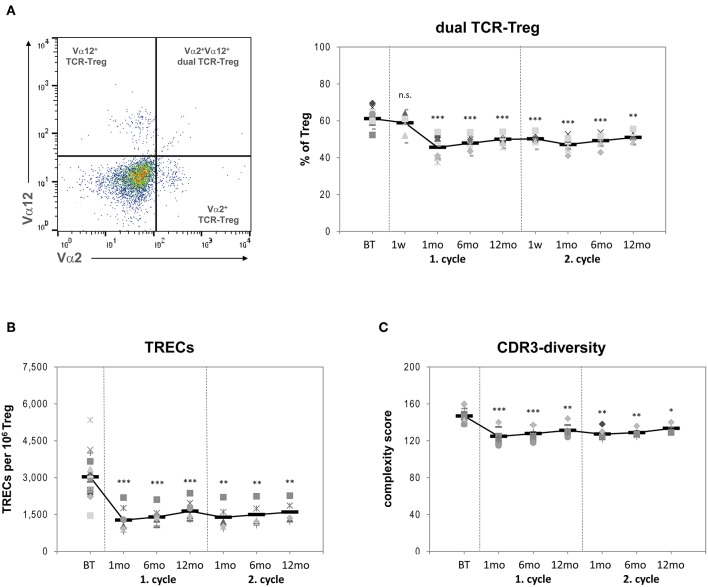
**(A)** Left side: Flow cytometry of stained PBMCs: After gating for Treg (see Figure 1) Vα2^+^, Vα12^+^, and Vα2Vα12-double positive cells were identified and used to calculate the frequencies of %dual-TCR Treg. Right side: Proportions of dual-TCR Treg within the total Treg population of 25 RRMS patients before treatment (BT) and up to 24 months after two cycles of alemtuzumab. **(B)** TREC numbers per 10^6^ Treg within the total Treg population of 25 RRMS patients BT and up to 24 months after two cycles of alemtuzumab. **(C)** CDR3 spectratyping complexity score of total Treg cells. Each symbol represents an individual patient. Means are indicated by bold line. Statistical significances in differences of each time point compared to BT as determined by two-tailed non-parametric one-sample Wilcoxon sign rank test are indicated (n.s. = non-significant; ^*^p < 0.05; ^**^p < 0.01; ^***^p < 0.001).

The post-depletional Treg population contained invariably decreased proportions of dual-TCR cells and markedly contracted TREC numbers compared with Treg assessed at baseline, and the decline in these thymic-dependent markers persisted throughout months 1–12 after each alemtuzumab cycle (Figures 2A,B; Table 1). Furthermore, as determined by CDR3 spectratyping post-treatment, Treg exhibited a reduced mean complexity score, reflecting a more pronouncedly constricted TCR repertoire than at baseline, a feature that lasted until month 12 of each treatment period (Figure 2C; Table 1). Altogether, these findings are consistent with the notion that homeostatic proliferation of cells that have escaped depletion by alemtuzumab, rather than meaningful release of newly generated cells from the thymus, dominates the recovery phase in the Treg compartment.

### Treg Suppressive Function

**Figure 3:**
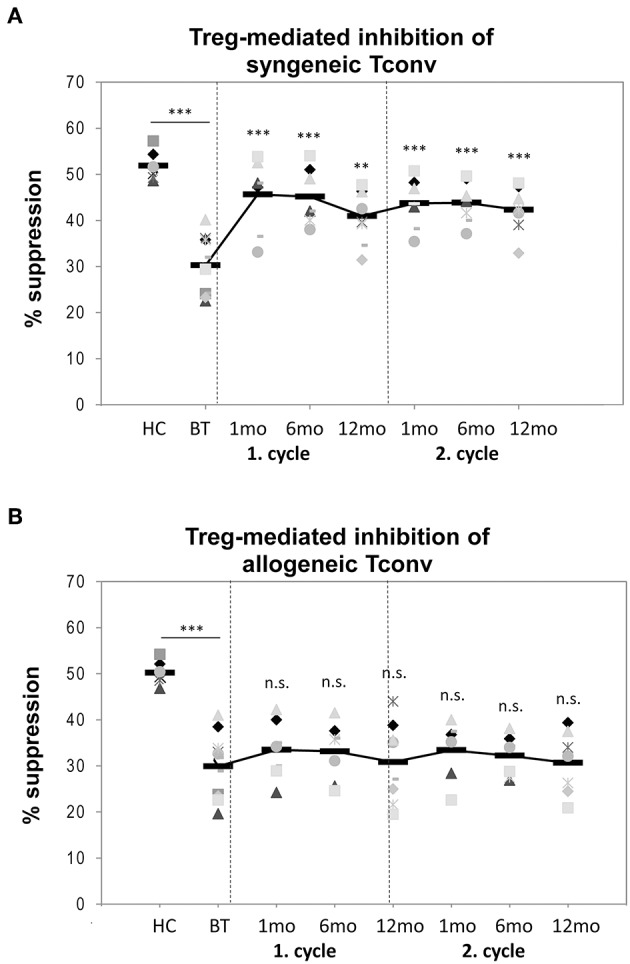
Treg suppressive function. Inhibitory capacities of Treg isolated from peripheral blood samples obtained from six healthy donors (HC) and from 22 RRMS patients before treatment (BT) and up to 24 months after two cycles of alemtuzumab as tested by *in vitro* proliferation assays against **(A)** syngeneic Tconv (black diamonds) or **(B)** allogeneic Tconv (gray cubes). Means are indicated by bold line. Statistical significances in differences of each time point compared to BT as determined by two-tailed non-parametric one-sample Wilcoxon sign rank test are indicated (n.s. = non-significant; ^**^p < 0.01; ^***^p < 0.001).

When tested in cocultures with syngeneic effector T-cells, the suppressive function of post-depletional Treg remained constantly increased vs. baseline over the entire study period (before treatment (BT): 30.2 ± 5.6% [mean percentage of Treg-mediated suppression of Tcon proliferation *in vitro*]; year 1: 1 month: 45.6 ± 6.8%, *p* < 0.001; 6 months: 45.1 ± 5.3%, *p* < 0.001; 1 year: 40.9 ± 5.2%, *p* < 0.01; year 2: 1 month: 43.7 ± 4.7%, *p* < 0.001; 6 months: 43.8 ± 4.0%, *p* < 0.001; 1 year: 42.3 ± 4.5%, *p* < 0.001). Thus, the inhibitory function of post-depletional Treg nearly reached levels comparable to those seen in Treg obtained from healthy donors (51.1 ± 3.3%; Figure 3A). In contrast, when co-cultured with allogeneic T-cells from normal donors, Treg inhibitory capacities remained more or less stable compared with their performance at baseline (BT: 29.9 ± 6.3%; year 1: 1 month: 33.5 ± 5.5%, *p* = 0.28; 6 months: 33.2 ± 5.5%, *p* = 0.33; 1 year: 30.8 ± 7.7%, *p* = 0.80; year 2: 1 month: 33.4 ± 5.6%, *p* = 0.29; 6 months: 32.2 ± 3.8%, *p* = 0.45; 1 year: 30.7 ± 6.0%, *p* = 0.82) and clearly reduced as compared to their counterparts from healthy control donors (50.2 ± 4.6%, *p* < 0.001; Figure 3B). Thus, repopulated Tcon obviously exhibited a decreased ability to resist regulation, thereby mitigating the MS-associated Treg defect previously described (18–22).

### Repopulation of CCR7^+^ and CCR7^−^ Tcon

**Figure 4:**
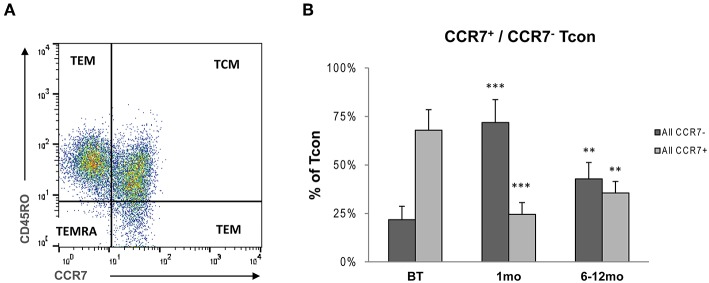
**(A)** Flow cytometry of stained PBMCs: After gating for Tcon cells were analyzed for surface expression of CCR7 identifying CCR7-expressing Tcon phenotypes (CCR7^+^CD45RO^+^ central memory cells, TCM; CCR7^+^CD45RO^−^ naive cells, TCRA) and CCR7^−^ Tcon subtypes (CCR7^−^CD45RO^+^ effector memory cells, TEM; CCR7^−^CD45RO^−^ cells, TEMRA). **(B)** Proportions of CCR7^+^ and CCR7^−^ subtypes within Tcon in peripheral blood samples obtained from eight RRMS patients before treatment (BT), and 1 month and 6–12 months after administration of alemtuzumab. Bars represent means with standard deviations. Statistical significances in differences of each time point compared to BT as determined by two-tailed non-parametric one-sample Wilcoxon sign rank test are indicated (^**^p < 0.01; ^***^p < 0.001).

It was reported that treatment with alemtuzumab affects the balance between CD4^+^ T cells either expressing or lacking the C-C chemokine receptor type 7 (CCR7) (11). CCR7 is involved in homing of T cells to various secondary lymphoid organs including lymph nodes (36). We could previously demonstrate that in fingolimod-treated MS patients CCR7-negative Tcon expand and are more prone to Treg mediated suppression when tested in *in vitro* proliferation assays, thereby indirectly upregulating Treg efficiency (30). We therefore analyzed frequencies of circulating CCR7^+^ and CCR7^−^ CD4^+^ T-cell subsets before and post treatment with alemtuzumab in a subcohort of eight patients in the 1st year post-alemtuzumab by flow cytometry (Figure 4A). At month 1, proportions of CCR7-expressing Tcon phenotypes (CCR7^+^CD45RO^+^ central memory cells, TCM; CCR7^+^CD45RO^−^ naive cells, TCRA) were massively decreased (BT: 67.9 ± 10.6% (mean percent of Tcon); 1 month 24.5 ± 6.1%, *p* < 0.0001) and were still reduced at 6 and12 months (35.6 ± 5.9%, *p* < 0.001). In contrast, the frequencies of CCR7^−^ Tcon subtypes (CCR7^−^CD45RO^+^ effector memory cells, TEM; CCR7^−^CD45RO^−^ cells, TEMRA) were expanded (BT: 21.8 ± 6.9%; 1 month: 71.9 ± 11.8%, *p* < 0.0001; 6–12 months: 42.8 ± 8.5%, *p* < 0.001) (Figure 4B; Table 1).

### CD52 Surface Expression and CD52-Mediated Cytolysis of Treg and Tcon

**Figure 5:**
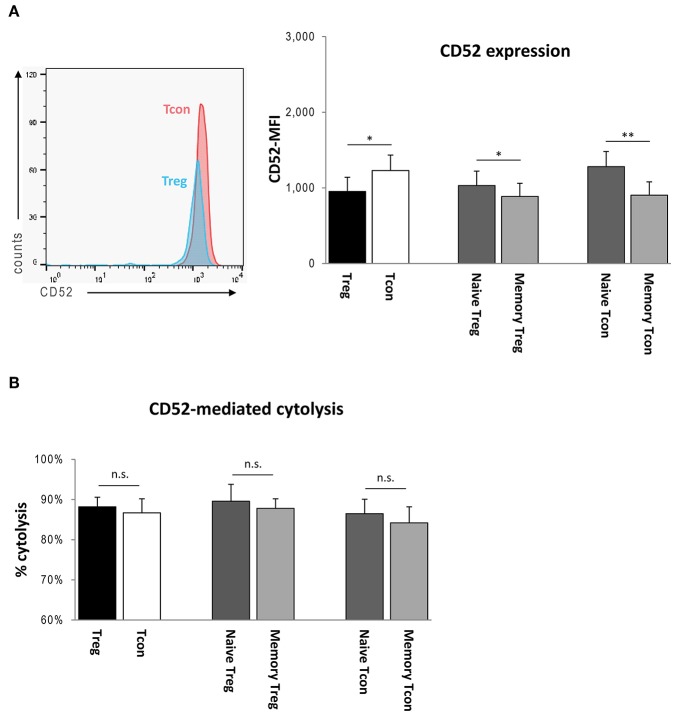
**(A)** Left side: Flow cytometry of stained PBMCs: After gating for Treg and Tcon, CD52 surface expressions were determined as mean fluorescence intensities (MFIs). Right side: CD52-MFIs of Treg, Tcon, and Treg and Tcon subsets obtained from peripheral blood samples of five HC. **(B)** CD52-mediated cytolysis of Treg, Tcon, and Treg and Tcon subsets isolated from peripheral blood samples of five HC as determined by a complement-dependent cytolysis assay *in vitro*. Bars represent means with standard deviations. Statistical significance as determined by Student's *t*-test is indicated (n.s. = non-significant; ^*^p < 0.05; ^**^p < 0.01).

Cellular CD52 expression on Treg, Tcon, and their subtypes was determined by flow cytometry of PBMCs obtained from five healthy donors. CD52 expression levels were higher on Tcon than on Treg (Tcon: MFI 1230 ± 203; Treg: 952 ± 188; *p* < 0.05), and were significantly higher on naïve vs. memory subsets (Treg_naive_ 1,030 ± 192, Treg_memory_ 886 ± 174, *p* < 0.05; Tcon_naive_ 1,281 ± 201, Tcon_memory_ 903 ± 177, *p* < 0.01) (Figure 5A).

To assess the effect of alemtuzumab on Treg and Tcon *in vitro*, freshly isolated Treg and Tcon obtained from five healthy donors were cultured alone for 3 h in the presence of 10 μg/ml alemtuzumab and 10% normal human complement. Cytolysis rates for each subtype were measured by flow cytometry as outlined in the methods section above, revealing that both Treg and Tcon were abundantly depleted in response to alemtuzumab (Treg: 88.2 ± 2.9% [proportion of PI-positive cells], Tcon: 86.7 ± 3.5%; *p* = 0.064; Figure 5B). Moreover, cells with a naïve phenotype contained in both CD4^+^ populations were depleted more efficiently, although the difference did not attain statistical significance (Treg_naive_ 89.6 ± 4.2%, Treg_memory_ 87.8 ± 5.1%, *p* = 0.101; Tcon_naive_ 86.5 ± 3.6%, Tcon_memory_ 84.2 ± 4.0%, *p* = 0.071; Figure 5B).

## Discussion

Alemtuzumab-induced lymphocyte depletion is followed by an asymmetric repopulation of circulating T- and B-cells which also impacts Treg. Previous studies have shown that Treg rapidly increase relative to other CD4^+^ T-cell subsets and remain enriched up to several months after therapy (10–14). It is obvious that such numeric changes between Treg and Tcon or alterations affecting the homeostatic composition of subtypes comprised in both compartments are of high clinical relevance, as Treg are important mediators in the maintenance of peripheral immunotolerance by suppressing potentially autoaggressive Tcon clones. In patients with MS, a more detailed understanding of alemtuzumab-triggered depletion and recovery in the Treg population is of particular interest, since MS patient-derived Treg are less potent inhibitors of Tcon responses as a result of altered prevalence of naïve and mature Treg subtypes compared to healthy controls (17–22).

Here, we found that Treg are lysed together with other CD4^+^ T-cells, as reflected by equivalent relative proportions of CD4^+^CD25^high^CD127^low^FOXP3^+^ cells contained in total CD4^+^ T-cells at baseline and at 1 week after alemtuzumab administration. Accordingly, when exposed to alemtuzumab and human complement *ex vivo*, Treg were destroyed at least as efficiently as Tcon despite the somewhat lower membrane expression of CD52 on Treg. Again, the small difference in surface densities of CD52 featured by naïve vs. memory subsets translated into a slightly higher susceptibility of both Treg_naive_ and Tcon_naive_ to undergo complement-mediated cell lysis compared with the respective memory counterparts. Hence, Treg and Treg subsets are not spared from the effects of alemtuzumab.

The peak expansion of Treg at month 1 during post-treatment lymphocyte recovery featured a substantial dominance of Treg_memory_ over Treg_naï*ve*_ and thus mirrored the dynamics of reconstituting memory and naïve phenotypes in CD4^+^ and CD8^+^ T-cells described earlier (10, 11). The imbalance between Treg_memory_ and Treg_naï*ve*_ persisted until month 12 after both alemtuzumab cycles and was accompanied by a continued deficit in relative frequencies of Treg_RTE_. As numbers of TREC-containing and dual TCR-Treg—both markers intrinsic to thymocytes—remained low throughout the study period and coincided with a compromised TCR repertoire as documented by a reduced CDR3 complexity score, these observations altogether confirm that thymopoiesis is not or barely induced after alemtuzumab therapy, possibly due to the premature decline in thymic function attributed to MS (19, 29, 30, 32).

Whether the abundance of cells with a Treg phenotype among post-depletional total CD4^+^ T-cells, detectable from month 1 through month 12 independent of the underlying disease, as shown in our study and former studies, is driven by Treg formed extrathymically in the peripheral immune system by expansion of residual Treg and/or conversion from memory Tcon (induced or iTreg) during treatment-induced homeostatic proliferation (10–14) remains elusive, since currently, no features or phenotypic markers, including the Ikaros family transcription factor Helios, are able to distinguish iTreg from Treg generated by homeostatic proliferation (37). However, we observed in occasional patients (*n* = 2, data not shown) that Treg obtained shortly after treatment are not highly proliferative, as depicted by barely detectable intracellular expression of Ki-67, a nuclear antigen exclusively expressed during active cell cycling (38). As similar results were reported in a previous study, where only one out of four individuals assessed after treatment with Campath-1 showed a post-depletional slight increase in active cycling of Treg (12), these findings support the notion that homeostatic expansion does not solely account for the increased Treg/Tcon ratio.

A predominance of Treg_memory_ negatively affects the immunoregulatory properties of total Treg, as we and others have shown previously (19, 23–25). We therefore sought to establish how post-alemtuzumab Treg and Tcon interact to promote the restored long-term suppressive function of Treg observed in a former study (11). Of note, despite the more pronounced imbalance in Treg_naive_ and Treg_memory_ proportions inherent to post-treatment cells, the suppressive performance of total Treg appeared unexpectedly raised throughout months 1–24 when tested against Tcon isolated from the same patient. When employing allogeneic instead of syngeneic Tcon, however, Treg activity *per se* remained stable vs. baseline and decreased compared with counterparts from healthy donors. This, in turn, clearly indicates that post-alemtuzumab Treg remain dysfunctional, but perform better as an indirect result of a treatment-induced redistribution in Tcon phenotypes. Accordingly, the post-treatment Tcon population turned out to be enriched in CCR7^−^ TEM and TEMRA cells at the expense of CCR7^+^ naive TCM and TCRA subsets. The preponderance of such effector memory cells parallels our own observations in fingolimod-treated patients with MS, where Tcon lacking CCR7 become elevated during treatment and are less proliferative, thereby indirectly upregulating Treg efficiency (30). Importantly, it has been recently suggested, that protracted T-cell recovery and, hence, long-lasting lack of T-cell regulation along with earlier and even hyperproliferative B-cell subset reconstitution might serve as a main driver in the emergence of secondary autoimmunity in response to alemtuzumab therapy (39). Whether homeostatically disturbed Treg/Tcon subsets favor breakthrough MS activity remains elusive. In this study, the disease unequivocally remained clinically stable in the early post-treatment phase, i.e., when the ratio of absolute numbers of Tcon and Treg was lowest (month 1), whereas the Tcon/Treg ratio of recovering lymphocytes had reached at least around 50% of that measured at baseline in all nine relapses documented. However, the numeric relation between Tcon and Treg did not significantly differ in stable patients when assessed at the same points in time.

## Conclusions

Taken together, Treg are not spared in alemtuzumab therapy and thymopoiesis does not considerably contribute to post-depletional long-term recovery. Reconstitution may be driven by homeostatic proliferation and/or by conversion from residual Tcon beginning after day 7 of treatment. This results in a relative Treg expansion among total CD4^+^ T-cells, which is, however, accompanied by a marked and long-lasting predominance of Treg_memory_ along with a contraction in Treg_naive_ and Treg_RTE_ as a response to therapeutic lymphopenia. As a result of these changes and the concomitant accumulation of effector memory populations in the Tcon compartment, the suppressive capacity of dysfunctional patient Treg is paradoxically restored. Importantly, intra-individual differences in repopulated Treg and Tcon may impact the therapeutic efficacy of alemtuzumab with respect to stabilization of MS activity. By showing that Treg undergo distinct and long-lasting homeostatic changes after alemtuzumab therapy, we challenge the beneficial influence deduced from the previously described apparent sparing of these cells from treatment-induced immune cell lysis (10, 11, 13).

## Ethics Statement

The protocol was approved by the University Hospital Heidelberg ethics committee; all individuals gave written informed consent.

## Author Contributions

JH and BW contributed to the conception and design of the study. JH, TR, AV, SP, SM, and MK-K performed research and contributed clinical samples. SJ organized the database. CW performed the statistical analysis. JH wrote the first draft of the manuscript. BW and SJ wrote sections of the manuscript. All authors contributed to the manuscript revision, and read and approved the submitted version.

### Conflict of Interest Statement

SM receives honoraria for lecturing, and travel expenses for attending meetings from Almirall, Amicus Therapeutics Germany, Bayer Health Care, Biogen, Celgene, Diamed, Genzyme, MedDay Pharmaceuticals, Merck Serono, Novartis, Novo Nordisk, ONO Pharma, Roche, Sanofi-Aventis, Chugai Pharma, QuintilesIMS, and Teva. His research is funded by the German Ministry for Education and Research (BMBF), Deutsche Forschungsgemeinschaft (DFG), Else Kröner Fresenius Foundation, German Academic Exchange Service, Hertie Foundation, Interdisciplinary Center for Clinical Studies (IZKF) Muenster, German Foundation Neurology and Almirall, Amicus Therapeutics Germany, Biogen, Diamed, Fresenius Medical Care, Genzyme, Merck Serono, Novartis, ONO Pharma, Roche, and Tevahas received honoraria for lecturing, travel reimbursements, and financial research support from Bayer, Biogen, Sanofi Genzyme, Merck Serono, Merck Sharp and Dohme, Novartis, Novo Nordisk, Sanofi Aventis, UCB and Teva. BW has received research grants and/or honoria from Merck Serono, Biogen, Teva, Novartis, Sanofi Genzyme, Bayer Healthcare, and research grants from the Dietmar Hopp Foundation, the Klaus Tschira Foundation and the Deutsche Forschungsgemeinschaft (DFG). None of the funding sources (mentioned in the acknowledgments) had a role in study design; collection, analysis, and interpretation of data; writing of the report; or the decision to submit the paper for publication. The remaining authors declare that the research was conducted in the absence of any commercial or financial relationships that could be construed as a potential conflict of interest.

## References

[1] Hartung HP Aktas O Boyko AN. Alemtuzumab: a new therapy for active relapsing-remitting multiple sclerosis. Mult Scler. (2015) 21:22–34. 10.1177/1352458514549398 25344374 PMC4361497

[2] Coles AJ Compston DA Selmaj KW Lake SL Moran S Margolin DH. Alemtuzumab vs. interferon beta-1a in early multiple sclerosis. N Engl J Med. (2008) 359:1786–801. 10.1056/NEJMoa0802670 18946064

[3] Coles AJ Twyman CL Arnold DL Cohen JA Confavreux C Fox EJ. Alemtuzumab for patients with relapsing multiple sclerosis after disease-modifying therapy: a randomised controlled phase 3 trial. Lancet. (2012) 380:1829–39. 10.1016/S0140-6736(12)61768-1 23122650

[4] Coles AJ Fox E Vladic A Gazda SK Brinar V Selmaj KW. Alemtuzumab more effective than interferon β-1a at 5-year follow-up of CAMMS223 clinical trial. Neurology. (2012) 78:1069–78. 10.1212/WNL.0b013e31824e8ee7 22442431

[5] Cohen JA Coles AJ Arnold DL Confavreux C Fox EJ Hartung HP. Alemtuzumab versus interferon beta 1a as first-line treatment for patients with relapsing-remitting multiple sclerosis: a randomised controlled phase 3 trial. Lancet. (2012) 380:1819–28. 10.1016/S0140-6736(12)61769-3 23122652

[6] Ruck T Bittner S Wiendl H Meuth SG. Alemtuzumab in multiple sclerosis: mechanism of action and beyond. Int J Mol Sci. (2015) 16:16414–39. 10.3390/ijms160716414 26204829 PMC4519957

[7] Hu Y Turner MJ Shields J Gale MS Hutto E Roberts BL. Investigation of the mechanism of action of alemtuzumab in a human CD52 transgenic mouse model. Immunology. (2009) 128:260–70. 10.1111/j.1365-2567.2009.03115.x 19740383 PMC2767316

[8] Krumbholz M Derfuss T Hohlfeld R Meinl E. B cells and antibodies in multiple sclerosis pathogenesis and therapy. Nat Rev Neurol. (2012) 8:613–23. 10.1038/nrneurol.2012.203 23045237

[9] Cox AL Thompson SA Jones JL Robertson VH Hale G Waldmann H. Lymphocyte homeostasis following therapeutic lymphocyte depletion in multiple sclerosis. Eur J Immunol. (2005) 35:3332–42. 10.1002/eji.200535075 16231285

[10] Zhang X Tao Y Chopra M Ahn M Marcus KL Choudhary N Differential reconstitution of T cell subsets following immunodepleting treatment with alemtuzumab (anti-CD52 monoclonal antibody) in patients with relapsing-remitting multiple sclerosis. J Immunol. (2013) 91:5867–74. 10.4049/jimmunol.1301926 24198283

[11] Jones JL Thompson SA Loh P Davies JL Tuohy OC Curry AJ. Human autoimmunity after lymphocyte depletion is caused by homeostatic T-cell proliferation. Proc Natl Acad Sci USA. (2013) 110:20200–5. 10.1073/pnas.1313654110 24282306 PMC3864306

[12] Bloom DD Chang Z Fechner JH Dar W Polster SP Pascual J. CD4+ CD25+ FOXP3+ regulatory T cells increase de novo in kidney transplant patients after immunodepletion with Campath-1H. Am J Transplant. (2008) 8:793–802. 10.1111/j.1600-6143.2007.02134.x 18261176

[13] Havari E Turner MJ Campos-Rivera J Shankara S Nguyen TH Roberts B. Impact of alemtuzumab treatment on the survival and function of human regulatory T cells in vitro. Immunology. (2014) 141:123–31. 10.1111/imm.12178 24116901 PMC3893855

[14] Rao SP Sancho J Campos-Rivera J Boutin PM Severy PB Weeden T. Human peripheral blood mononuclear cells exhibit heterogeneous CD52 expression levels and show differential sensitivity to alemtuzumab mediated cytolysis. PLoS ONE. (2012) 7:e39416. 10.1371/journal.pone.0039416 22761788 PMC3382607

[15] Sakaguchi S Sakaguchi N Shimizu J Yamazaki S Sakihama T Itoh M. Immunologic tolerance maintained by CD25+ CD4+ regulatory T cells: their common role in controlling autoimmunity, tumor immunity, and transplantation tolerance. Immunol Rev. (2001) 182:18–32. 10.1034/j.1600-065X.2001.1820102.x 11722621

[16] Sakaguchi S Sakaguchi N Asano M Itoh M Toda M Immunologic self-tolerance maintained by activated T cells expressing IL-2 receptor a-chains (CD25). Breakdown of a single mechanism of self-tolerance causes various autoimmune diseases. J Immunol. (1995) 155:1151–64. 7636184

[17] Viglietta V Baecher-Allan C Weiner HL Hafler DA. Loss of functional suppression by CD4+CD25+ regulatory T cells in patients with multiple sclerosis. J Exp Med. (2004) 199:971–9. 10.1084/jem.20031579 15067033 PMC2211881

[18] Haas J Hug A Viehöver A Fritzsching B Falk CS Filser A. Reduced suppressive effect of CD4+CD25high regulatory T cells on the T cell immune response against myelin oligodendrocyte glycoprotein in patients with multiple sclerosis. Eur J Immunol. (2005) 35:3343–52. 10.1002/eji.200526065 16206232

[19] Haas J Fritzsching B Trübswetter P Korporal M Milkova L Fritz B. Prevalence of newly generated naive regulatory T cells (Treg) is critical for Treg suppressive function and determines Treg dysfunction in multiple sclerosis. J Immunol. (2007) 179:1322–30. 10.4049/jimmunol.179.2.1322 17617625

[20] Venken K Hellings N Hensen K Rummens JL Medaer R D'hooghe MB. Secondary progressive in contrast to relapsing-remitting multiple sclerosis patients show a normal CD4+CD25+ regulatory T-cell function and FOXP3 expression. J Neurosci Res. (2006) 83:1432–46. 10.1002/jnr.20852 16583400

[21] Venken K Hellings N Thewissen M Somers V Hensen K Rummens JL. Compromised CD4+ CD25(high) regulatory T-cell function in patients with relapsing-remitting multiple sclerosis is correlated with a reduced frequency of FOXP3-positive cells and reduced FOXP3 expression at the single-cell level. Immunology. (2008) 123:79–89. 10.1111/j.1365-2567.2007.02690.x 17897326 PMC2433271

[22] Venken K Hellings N Broekmans T Hensen K Rummens JL Stinissen P. Natural naive CD4+CD25+CD127low regulatory T cell (Treg) development and function are disturbed in multiple sclerosis patients: recovery of memory Treg homeostasis during disease progression. J Immunol. (2008) 180:6411–20. 10.4049/jimmunol.180.9.6411 18424765

[23] Duszczyszyn DA Williams JL Mason H Lapierre Y Antel J Haegert DG. Thymic involution and proliferative T-cell responses in multiple sclerosis. J Neuroimmunol. (2010) 221:73–80. 10.1016/j.jneuroim.2010.02.005 20223525

[24] Balint B Haas J Schwarz A Jarius S Fürwentsches A Engelhardt K. T-cell homeostasis in pediatric multiple sclerosis. Old cells in young patients. Neurology. (2013) 81:1–9. 10.1212/WNL.0b013e3182a2ce0e 23911752

[25] Schwarz A Schumacher M Pfaff D Schumacher K Jarius S Balint B. Fine-tuning of regulatory T cell function: the role of calcium signals and naive regulatory T cells for regulatory T cell deficiency in multiple sclerosis. J Immunol. (2013) 190:4965–70. 10.4049/jimmunol.1203224 23576680

[26] Polman CH Reingold SC Banwell B Clanet M Cohen JA Filippi M. Diagnostic criteria for multiple sclerosis: 2010 Revisions to the McDonald criteria. Ann Neurol. (2011) 69:292–302. 10.1002/ana.22366 21387374 PMC3084507

[27] Haas J Korporal M Balint B Fritzsching B Schwarz A Wildemann B. Glatiramer acetate improves regulatory T-cell function by expansion of naïve CD4(+)CD25(+)FOXP3(+)CD31(+) T-cells in patients with multiple sclerosis. J Neuroimmunol. (2009) 216:113–7. 10.1016/j.jneuroim.2009.06.011 19646767

[28] Korporal M Haas J Balint B Fritzsching B Schwarz A Moeller S. Interferon beta-induced restoration of regulatory T-cell function in multiple sclerosis is prompted by an increase in newly generated naïve regulatory T cells. Arch Neurol. (2008) 65:1434–9. 10.1001/archneur.65.11.1434 19001161

[29] Haas J Korporal M Schwarz A Balint B Wildemann B. The interleukin-7 receptor α chain contributes to altered homeostasis of regulatory T cells in multiple sclerosis. Eur J Immunol. (2011) 41:845–53. 10.1002/eji.201041139 21287555

[30] Haas J Schwarz A Korporal-Kunke M Jarius S Wiendl H Kieseier BC Fingolimod does not impair T-cell release from the thymus and beneficially affects Treg function in patients with multiple sclerosis. Mult Scler. (2015) 21:1521–32. 10.1177/1352458514564589 25583847

[31] Tuovinen H Salminen JT Arstila TP. Most human thymic and peripheral-blood CD4+ CD25+ regulatory T cells express 2 T-cell receptors. Blood. (2006) 108:4063–70. 10.1182/blood-2006-04-016105 16926292

[32] Hug A Korporal M Schröder I Haas J Glatz K Storch-Hagenlocher B. Thymic export function and T cell homeostasis in patients with relapsing remitting multiple sclerosis. J Immunol. (2003) 171:432–7. 10.4049/jimmunol.171.1.432 12817027

[33] Genevée C Diu A Nierat J Caignard A Dietrich PY Ferradini L An experimentally validated panel of subfamily-specific oligonucleotide primers (V alpha 1-w29/Vbeta 1-w24) for the study of human T cell receptor variable V gene segment usage by polymerase chain reaction. Eur J Immunol. (1992) 22:1261–9. 10.1002/eji.1830220522 1533591

[34] Choi YW Kotzin B Herron L Callahan J Marrack P Kappler J. Interaction of Staphylococcus aureus toxin superantigens with human T cells. Proc Natl Acad Sci USA. (1989) 86:8941–5. 10.1073/pnas.86.22.8941 2479030 PMC298406

[35] Gorski J Yassai M Zhu X Kissela B Kissella B Keever C. Circulating T cell repertoire complexity in normal individuals and bone marrow recipients analyzed by CDR3 size spectratyping: correlation with immune status. J Immunol. (1994) 152:5109–19. 8176227

[36] Sharma N Benechet AP Lefrançois L Khanna KM. CD8 T cells enter the splenic T cell zones independently of CCR7, but the subsequent expansion and trafficking patterns of effector T cells after infection are dysregulated in the absence of CCR7 migratory cues. J Immunol. (2015) 195:5227–36. 10.4049/jimmunol.1500993 26500349 PMC4655190

[37] Burocchi A Colombo MP Piconese S. Convergences and divergences of thymus- and peripherally derived regulatory T cells in cancer. Front Immunol. (2013) 4:1–16. 10.3389/fimmu.2013.00247 23986759 PMC3753661

[38] Sieg SF Bazdar DA Lederman MM. Impaired TCR-mediated induction of Ki67 by naive CD4+ T cells is only occasionally corrected by exogenous IL-2 in HIV-1 infection. J Immunol. (2003) 171:5208–14. 10.4049/jimmunol.171.10.5208 14607921

[39] Baker D Herrod SS Alvarez-Gonzalez C Giovannoni G Schmierer K. Interpreting lymphocyte reconstitution data from the pivotal phase 3 trials of alemtuzumab. JAMA Neurol. (2017) 74:961–9. 10.1001/jamaneurol.2017.0676 28604916 PMC5710323

