# Supplementary figures and images for: Alemtuzumab in Multiple Sclerosis: Short- and Long-Term Effects of Immunodepletion on the Peripheral Treg Compartment
Source: Front Immunol. 2019 Jun 4;10:1204. doi: 10.3389/fimmu.2019.01204 (PMC6558003; doi:10.3389/fimmu.2019.01204)

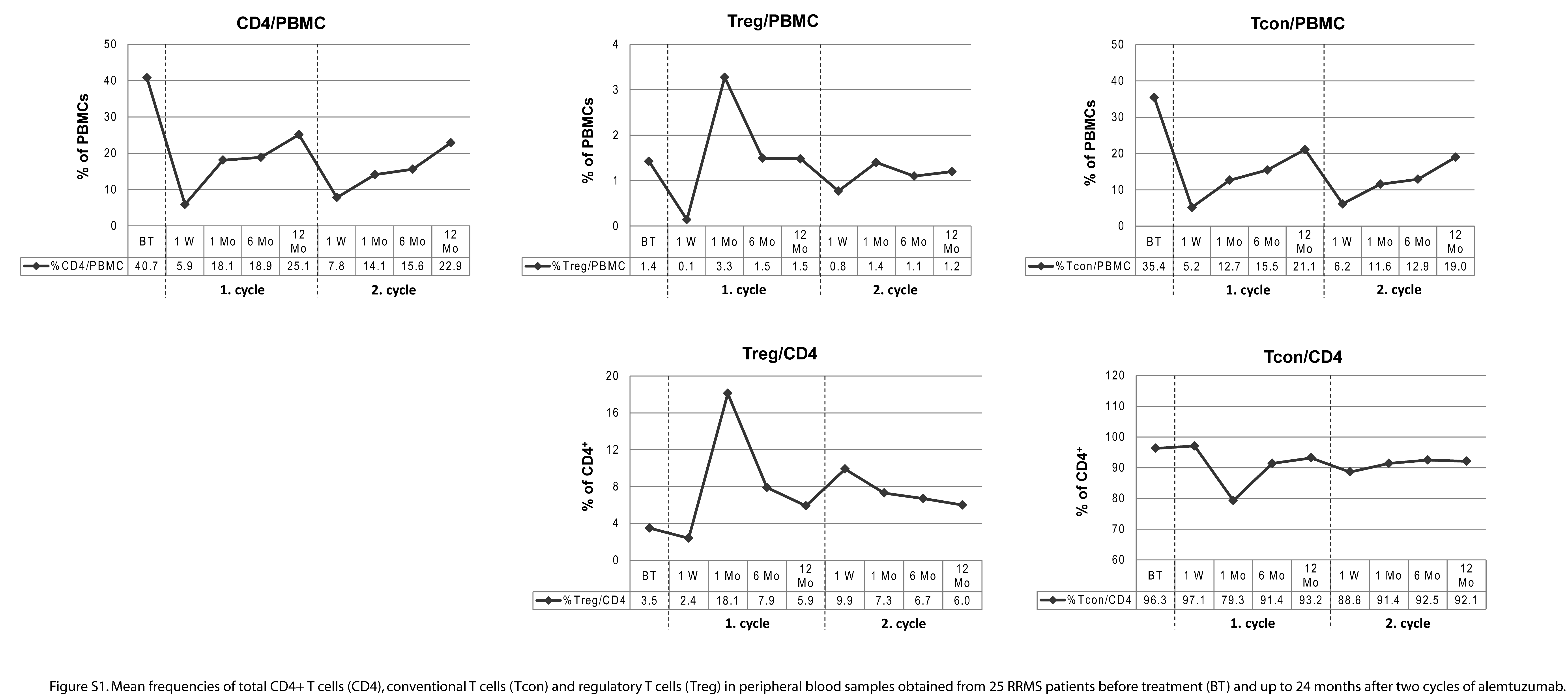

Supplement: Supplementary file 1 [file Image_1.TIF]

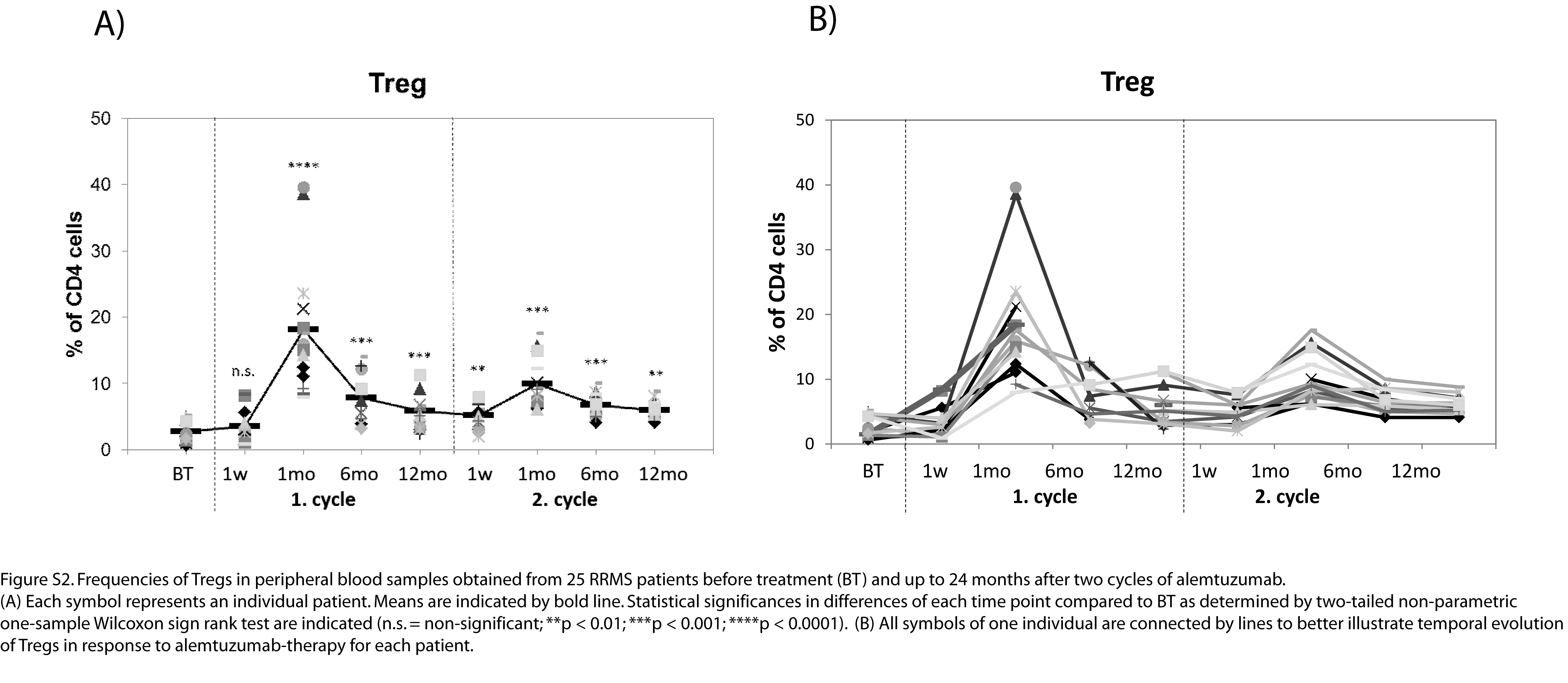

Supplement: Supplementary file 2 [file Image_2.TIF]
